# Editorial: *Dictyostelium*: A Tractable Cell and Developmental Model in Biomedical Research

**DOI:** 10.3389/fcell.2022.909619

**Published:** 2022-04-26

**Authors:** Robert J. Huber, Robin SB Williams, Annette Müller-Taubenberger

**Affiliations:** ^1^ Department of Biology, Trent University, Peterborough, ON, Canada; ^2^ Centre for Biomedical Sciences, School of Biological Sciences, Royal Holloway University of London, Egham, United Kingdom; ^3^ Department of Cell Biology (Anatomy III), Biomedical Center (BMC), LMU Munich, Munich, Germany

**Keywords:** cancer, cell biology, cell motility and chemotaxis, cell signalling, development, model system, neurological disease, pharmacogenetics screens

**FIGURE 1:**
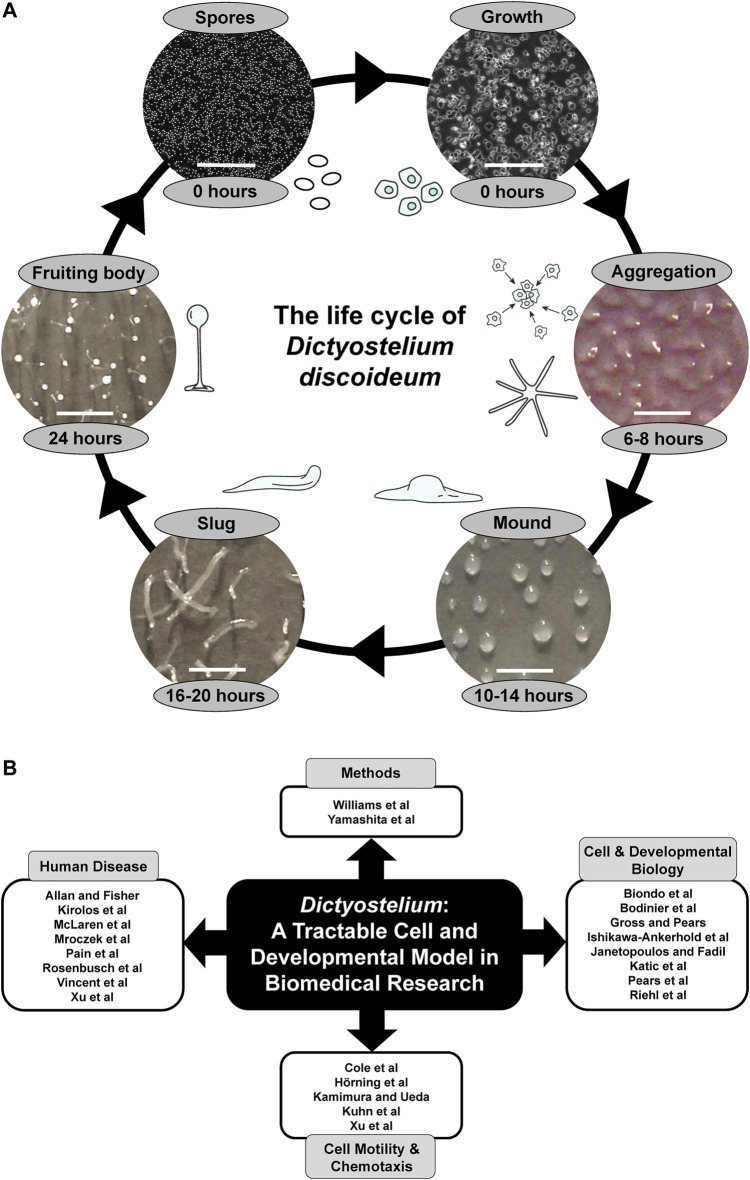
**(A)** The life cycle of *Dictyostelium discoideum*. During the growth phase, haploid amoebae consume nutrients and divide mitotically. Starvation initiates a 24-h developmental program that begins with the chemotactic aggregation of cells to form multicellular mounds, which is followed by a series of morphological events that generate motile slugs. The final phase of development involves terminal differentiation of cells and the formation of fruiting bodies composed of a mass of viable spores that rest atop a slender stalk. When nutrients become available, the spores germinate to restart the life cycle. White scale bars: 0.2 mm (spores and growth), 1 mm (aggregation, mound, slug, fruiting body). Some of the illustrations and microscopy images depicting the different life cycle stages were previously published in Huber, 2021 (permission provided by CC-BY license). **(B)** Themes of this Research Topic. Articles within this Research Topic are grouped into four main categories: Methods, Cell and Developmental Biology, Cell Motility and Chemotaxis, and Human Disease.

For almost a century, the social amoeba *Dictyostelium discoideum* has been used as an inexpensive and high-throughput model system for studying a variety of fundamental cellular and developmental processes including cell movement, chemotaxis, differentiation, and autophagy (Müller-Taubenberger et al., 2013; Mathavarajah et al., 2017). The life cycle of *Dictyostelium* is comprised of a unicellular growth phase and a 24-h multicellular developmental phase with distinct stages (Figure 1A). *Dictyostelium* development shares many common features with metazoan development but occurs in a much shorter time frame, which allows for the rapid detection of developmental phenotypes. The fully sequenced, low redundancy genome of *Dictyostelium* provides a less complex system to work with, whilst still maintaining many genes and related signalling pathways found in more complex eukaryotes (Eichinger et al., 2005). The *Dictyostelium* genome is haploid, which allows researchers to introduce one or multiple gene disruptions with relative ease, and gene function can be studied in a true multicellular organism with measurable phenotypic outcomes (Kuspa, 2006; Faix et al., 2013; Friedrich et al., 2015; Sekine et al., 2018). In addition, insertional mutant libraries facilitate pharmacogenetics screens that have enhanced our understanding of the function of bioactive compounds at a cellular level (Damstra Oddy et al., 2021; Warren et al., 2020). Finally, a variety of expression constructs are available that enable studies on protein localization and function in *Dictyostelium* (Levi et al., 2000; Veltman et al., 2009; Müller-Taubenberger and Ishikawa-Ankerhold, 2013).

More recently, *Dictyostelium* has emerged as a valuable biomedical model system for studying several human diseases. The genome encodes orthologs of genes associated with human disease and the signalling pathways that regulate the behaviour of *Dictyostelium* cells are remarkably similar to those observed in mammalian cells, which has allowed findings from *Dictyostelium* to be successfully translated to mammalian systems (Alexander and Alexander, 2011; Chang et al., 2012). As a result, *Dictyostelium* has, and will continue to offer, excellent opportunities to advance biomedical research.

This Research Topic contains 23 articles that showcase the use of *Dictyostelium* as a tractable cell, molecular, and developmental model system in biomedical research, and includes two methods articles that enhance the biomedical applications of this valuable model organism (Figure 1B). Yamashita et al. describe the application of CRISPR-based gene disruption in *Dictyostelium*, while Williams et al. report the development of a new positive selection high throughput genetic screen.

The use of *Dictyostelium* as a model system for studying fundamental cellular and developmental processes is well established and this Research Topic contains several articles describing new findings on conserved processes in *Dictyostelium* with biomedical relevance (Figure 1B). In their original research article, Bodinier et al. reveal a mechanism regulated by the leucine-rich repeat kinase LrrkA that facilitates the sensing, phagocytosis, and killing of bacteria by *Dictyostelium* amoebae. In addition, Biondo et al. describe how *Dictyostelium* can be used as a model system for studying aerotaxis, Riehl et al. propose a role for UBX domain-containing protein nine in protein homeostasis in *Dictyostelium*, and Ishikawa-Ankerhold et al. reveal the role of pH in cytoplasmic rod formation in *Dictyostelium*, which has implications for human diseases caused by actin-cofilin rod formation. In review articles, Katic et al. summarize the roles of dynamin superfamily proteins in regulating vesicular trafficking and host-pathogen interactions in *Dictyostelium*, Janetopoulos and Fadil review the role of PIP_2_ in regulating the localization and exocytosis of the contractile vacuole system in migrating *Dictyostelium* amoebae, and Pears et al. describe DNA repair mechanisms in *Dictyostelium*, and how further study of DNA repair in *Dictyostelium* can help us better understand how this process is dysregulated in cancer. Finally, in their Hypothesis and Theory article, Gross and Pears discuss how *Dictyostelium* can be used to study the roles of the nutrient and energy sensors, mTORC1 and AMPK, which are linked to several human diseases including Alzheimer’s disease, cancer, obesity, and type 2 diabetes.

Historically, *Dictyostelium* has been used as a model system to elucidate the signalling pathways regulating eukaryotic cell motility and chemotaxis, which has furthered our understanding of the mechanisms regulating cancer cell movement. This Research Topic includes several exciting articles highlighting recent advancements in this area (Figure 1B). For example, in their original research article, Hörning et al. describe the dynamics of actin polymerization and PIP_3_ activity in amoeboid cells. In addition, Xu et al. reveal the molecular mechanism and biological function of C2GAP1 membrane targeting for chemotaxis, and Cole et al. show the roles of actin-binding proteins in sensing and transmitting mechanical stimuli that drive directed cell migration. To supplement these original research articles, Kamimura and Ueda review studies in *Dictyostelium* that have helped elucidate the role of G protein-coupled receptor signalling in regulating eurkaryotic chemotaxis, and Kuhn et al. summarize recent advances in imaging, synthetic biology, and computational analysis that have allowed researchers to tune the activity of individual molecules in cells and precisely measure the effects on cellular motility and signalling.

A central theme that emerges from this Research Topic is the use of *Dictyostelium* as a model system for studying specific human diseases (Figure 1B). In their original research article, McLaren et al. knock out the *Dictyostelium* ortholog of human ceroid lipofuscinosis neuronal 5 (*CLN5*) and show that loss of the gene impacts growth and multicellular development by affecting autophagy. In humans, mutations in *CLN5* cause a subtype of Batten disease, the most common form of childhood neurodegeneration. This Research Topic also reports the ability of *Dictyostelium* to further our understanding of Parkinson’s disease. For example, in their original research article, Rosenbusch et al. report findings from *Dictyostelium* that link mutations in Parkinson’s disease-associated genes to aberrant mitochondrial activity. In addition, Mroczek et al. present data that improve our understanding of the interactions and cytotoxicity of tau and alpha-synuclein, both of which are linked to Parkinson’s disease. Further supporting the use of *Dictyostelium* as a model system for studying neurological disease, Vincent et al. review work in *Dictyostelium* and yeast that has provided insight into the roles of WIPI proteins in neurodegeneration, and Allan and Fisher characterize a new *Dictyostelium* model for the lysosomal storage disease, mucolipidosis type IV. This Research Topic also contains articles related to other human diseases. In their review, Pain et al. describe work in *Dictyostelium* that studied the role of decanoic acid in ketogenic diets, which have been used in the treatment of epilepsy, bipolar disorder, cancer, and diabetes. In addition, Kirolos et al. review work in *Dictyostelium* that has enhanced our understanding of the pathology underlying acute respiratory distress syndrome, and Xu et al. summarize studies in *Dictyostelium* that have provided insight into how phagocytes use chemotaxis and phagocytosis to detect and kill pathogens.

In total, this Research Topic showcases exciting new research in *Dictyostelium* biology that strengthen its position as a tractable cellular and developmental model system in biomedical research. However, we offer here only a sample of the many facets of medically relevant research currently performed using *Dictyostelium*. Therefore, we encourage readers to seek out additional literature from the field that has used *Dictyostelium* as a biomedical model system to learn more about its valuable contributions to human disease research.
